# An Age‐Adapted Co‐Design Methodology for Community Health Research Involving Older Adults With Type 2 Diabetes

**DOI:** 10.1111/hex.70718

**Published:** 2026-06-07

**Authors:** Jiangpan Niu, Yuanyuan Yin, Shan Wang

**Affiliations:** ^1^ Winchester School of Art University of Southampton Winchester UK

**Keywords:** co‐design, community health research, older adults, qualitative methodology, Type 2 diabetes

## Abstract

**Introduction:**

Co‐design is increasingly used in health and social care research, but its application with older adults in community settings may raise methodological challenges. Age‐adapted co‐design methods are needed to support meaningful participation.

**Methods:**

This study developed and examined an age‐adapted co‐design methodology in a community setting involving older adults with Type 2 diabetes mellitus (T2DM). A qualitative approach was adopted, which included exploratory interviews, design probes, clarification interviews, a co‐design workshop and evaluation interviews. A total of 24 participants were involved across the study, including older adults with T2DM, community workers, healthcare professionals and extended reality (XR) designers. The methodology was examined in terms of acceptability, appropriateness and feasibility.

**Results:**

The methodology was generally found to be acceptable, appropriate and feasible. Participants described the interviews, design probes and workshop activities as manageable and low‐burden, particularly when tasks drew on everyday life. Visual materials such as stickers, icons, images and videos, together with simple prompts and reduced writing demands, supported understanding of the research activities, expression and positive emotional engagement. The study also identified challenges related to unfamiliar co‐design concepts, the interpretation of some visual tools, workshop duration and practical implementation constraints such as staffing, space, funding, health conditions and time availability. Participant feedback and researcher reflection informed iterative refinements to the materials, workshop preparation, communication strategies and activity format during the study.

**Conclusion:**

This study demonstrates the value of an age‐adapted, multi‐stage co‐design methodology for engaging older adults in community health research. The findings highlight the importance of grounding co‐design in lived experience, using age‐friendly materials and refining methods iteratively.

**Patient or Public Contribution:**

Older adults with T2DM, community workers, healthcare professionals and XR designers contributed to this study. Their experiences and feedback informed the refinement of the research materials, co‐design activities and methodological process.

**Abbreviations**
EBCD: experience‐based co‐design
T2DM: Type 2 diabetes mellitus
XR: extended reality

## 1 Introduction

Co‐design is a collaborative methodology that actively involves people directly affected by an issue, place or process in its design and, in some cases, its implementation [1]. In health and social care, it can generate insider knowledge [2], improve service feasibility and value, and support more realistic and actionable feedback from multiple perspectives [3, 4]. It has also been linked to stronger collaboration, improved service accessibility and satisfaction in healthcare [5], reflecting a shift from designing for users to designing with patients and the public [6, 7]. This orientation is particularly relevant in chronic disease management, where effective self‐management is often shaped by physical, informational, financial and psychosocial barriers [8, 9]. Community‐based support is especially important because it is more accessible [10], is associated with better quality of life among older adults [11] and can improve health and care outcomes [12]. While many interventions still rely on hospital‐ or family‐centred models [13, 14], these approaches have become increasingly limited in the context of population ageing and growing chronic disease management needs [15]. By contrast, community health management has been described as a more sustainable alternative [16, 17]. Co‐design has already been applied in related chronic disease settings, including diabetes risk reduction [18], parkinson's self‐management [19] and culturally adapted diabetes support [20]. It has also contributed to closer collaboration in community health management [21].

However, applying co‐design with older adults also presents methodological challenges. Older adults may face communication, health and participation barriers [7]. Co‐design processes may become tokenistic, reproduce marginalisation [4] or reinforce power imbalances when older adults' agency and diversity are not adequately recognised [6]. In community and public organisations, co‐design may also be constrained by bureaucracy, established procedures and the coordination of multiple stakeholder interests [22, 23]. In digital contexts, participation can be further affected by differences in digital familiarity, concerns about technological change [24] and cognitive impairment [25]. Recent methodological work therefore emphasises that co‐design with older adults requires careful ethical and methodological adaptation, including expectation‐setting, facilitation [26] and the use of more personalised and accessible materials [27].

A range of qualitative, participatory and service‐oriented methods has been used to understand health experiences and support co‐design in practice. Interviews and focus groups can generate important insights into participants' views, experiences [28]. However, interviews may privilege individual‐level explanation [29], and focus groups do not always allow equal participation [30]. Participatory approaches such as experience‐based co‐design (EBCD) [31] and service design tools, including service blueprints [32] and user journey mapping [33], have also been applied in healthcare to support service improvement and identify patient experience issues [34, 35]. At the same time, their use with older adults may still be shaped by age, health, technology and cognitive constraints [24, 25]. Some tools may also require substantial organisational input [36] or remain limited to the user perspective unless combined with deeper analysis of back‐stage processes and resources [3]. Design probes offer another way to explore everyday lived experience by supporting reflection and self‐expression [37], as well as low‐pressure participation over time [38]. They also require age‐appropriate adaptation when used with older adults [39]. Such adaptations often involve clearer guidance, reduced participation demands and more flexible forms of expression to make participation more manageable and accessible for older adults.

Overall, these studies suggest that although various qualitative, participatory and service‐oriented methods have been used in health research, no single method is sufficient to capture the complexity of older adults' everyday health management in community settings. What remains lacking is a structured, age‐adapted co‐design methodology that supports participation across sequential stages while remaining grounded in lived experience. This study therefore develops and empirically examines such a methodology for community health research with older adults with chronic conditions. By integrating exploratory interviews, design probes, clarification interviews, co‐design workshops and evaluation interviews, it explores how a staged process can support accessibility, engagement and contextually grounded insights. The methodology is evaluated in terms of acceptability, appropriateness and feasibility [40].

## 2 Methodology

### 2.1 Study Design

This study reports the methodological component of a broader community‐based extended reality (XR) exercise study involving older adults with Type 2 diabetes mellitus (T2DM). It adopted a qualitative design to develop and examine an age‐adapted co‐design methodology for community‐based health research involving older adults with T2DM. A co‐design orientation allowed participants' experiences and feedback to inform the refinement of the research process [41]. The study focused on the methodology itself, particularly its acceptability, appropriateness and feasibility in real‐world practice, rather than on health or behavioural outcomes.

### 2.2 Methodological Overview

To address the methodological challenges of engaging older adults with chronic conditions in co‐design health research, this study developed and applied an age‐adapted co‐design methodology. It comprised five sequential stages: (1) exploratory interviews, (2) design probes, (3) clarification interviews, (4) co‐design workshop and (5) evaluation interviews. This sequence moved from individual experience exploration to collaborative idea generation and evaluation. The five‐stage process was adapted to the communication styles, participation capacities and everyday lives of older adults in community settings. It aimed to reduce participation burden, support gradual familiarisation with research activities and ensure that later co‐design stages were grounded in participants' lived experiences rather than abstract discussion alone.

### 2.3 Setting and Sample Strategy

The study was conducted in an urban community in Liwan District, Guangzhou, China. It focused on community‐based exercise engagement and the co‐design of XR‐supported health service ideas among older adults with T2DM. Participants included older adults with T2DM, community workers, healthcare professionals and XR designers. These groups took part in different stages of the study according to their roles and expertise. Older adults provided lived experience, while community workers, healthcare professionals and XR designers offered implementation, clinical and design perspectives. Ethical approval was obtained from the University of Southampton Research Ethics Committee, and all participants received written and verbal information about the study and provided informed consent.

Eligible older adult participants were required to meet the following criteria: (1) be aged 60 years or above; (2) have a previous diagnosis of T2DM; (3) live in the community; (4) be able to communicate in Mandarin or with bilingual support, and (5) be cognitively able to provide informed consent and participate. Individuals with severe cognitive impairment, severe visual or hearing loss that affected communication or those undergoing institutional or hospital‐based rehabilitation were excluded.

Participants were recruited through community recommendations, voluntary sign‐ups and peer referrals. Older adult participants were approached mainly through community networks and local recommendations. Community workers were recruited with the assistance of the community leader, who helped identify staff members involved in organising or supporting health‐ and exercise‐related activities for older adults. Healthcare professionals were recruited through the community hospital linked to the same community and through university‐based medical contacts. XR designers were recruited through design research networks and personal or professional referrals, followed by direct invitation.

In qualitative methodological research, sampling is guided not by statistical representativeness but by the need to obtain rich, relevant and diverse perspectives that can inform the research aims in depth [42, 43]. A purposive sampling strategy was adopted to recruit information‐rich participants whose lived experiences, professional roles or design expertise aligned closely with the methodological focus of the study. Attention was also given to demographic and experiential diversity within and across stakeholder groups [44, 45]. Snowball sampling was applied where necessary to recruit harder‐to‐access stakeholder groups [46]. Sample adequacy was judged in relation to the diversity of stakeholder perspectives, the richness of the multi‐stage data and the recurrence of key methodological patterns across stages. In qualitative research, saturation marks the point at which no new, relevant information arises and signals the conclusion of sampling [47, 48]. In this study, attention was therefore given to whether new information relevant to the study aims continued to emerge across stakeholder groups and sequential stages of data collection. Transportation reimbursement and small appreciation gifts were provided to acknowledge participants' time and contribution.

Sample size in qualitative research is shaped by the scope, purpose and complexity of the study rather than by statistical representativeness [49, 50]. The participant pool comprised 24 individuals across four stakeholder groups [51, 52]. This structure was designed to balance manageability with diversity while capturing the key experiential, professional and design perspectives needed to examine the methodology across the sequential stages of the study. A total of 20 participants were involved in the first stage, including 8 older adults with T2DM, 6 community workers, 4 healthcare professionals and 2 XR designers. All eight older adults continued into the design probe stage, and one later withdrew before the clarification interview stage due to time and health constraints, leaving seven participants. The co‐design workshop and follow‐up evaluation stages involved eight participants, including both participants from earlier stages and four newly recruited participants. These later‐stage additions comprised two older adults with T2DM, one community worker and one healthcare professional, and were included to support the requirements of the collaborative and evaluative stages of the sequential design. Older adult participants were aged 60–78 years.

### 2.4 Methods in Practice

Data collection was conducted across five stages using semi‐structured interviews, design probes, clarification interviews, a co‐design workshop and evaluation interviews. Together, these stages generated interview transcripts, probe records and workshop materials that captured both individual experiences and collaborative reflections.

#### 2.4.1 Stage 1: Exploratory Semi‐Structured Interviews

Semi‐structured interviews were used first because they are well suited to situations where little prior research exists and where participants may need space to discuss personal experiences, beliefs and motivations in detail, including issues that may be less comfortable to raise in group settings [53, 54]. The interviews addressed exercise behaviours, barriers and facilitators to participation, emotional experiences related to health management and expectations for digital or community‐based exercise support, including preferences for tracking and feedback. This stage involved 20 participants across four stakeholder groups: 8 older adults with T2DM, 6 community workers, 4 healthcare professionals and 2 XR design practitioners. Each interview lasted approximately 30–60 min and was audio‐recorded with consent. The findings provided an initial understanding of exercise engagement in context and informed the design of the probe materials.

#### 2.4.2 Stage 2: Design Probes

**Figure 1:**
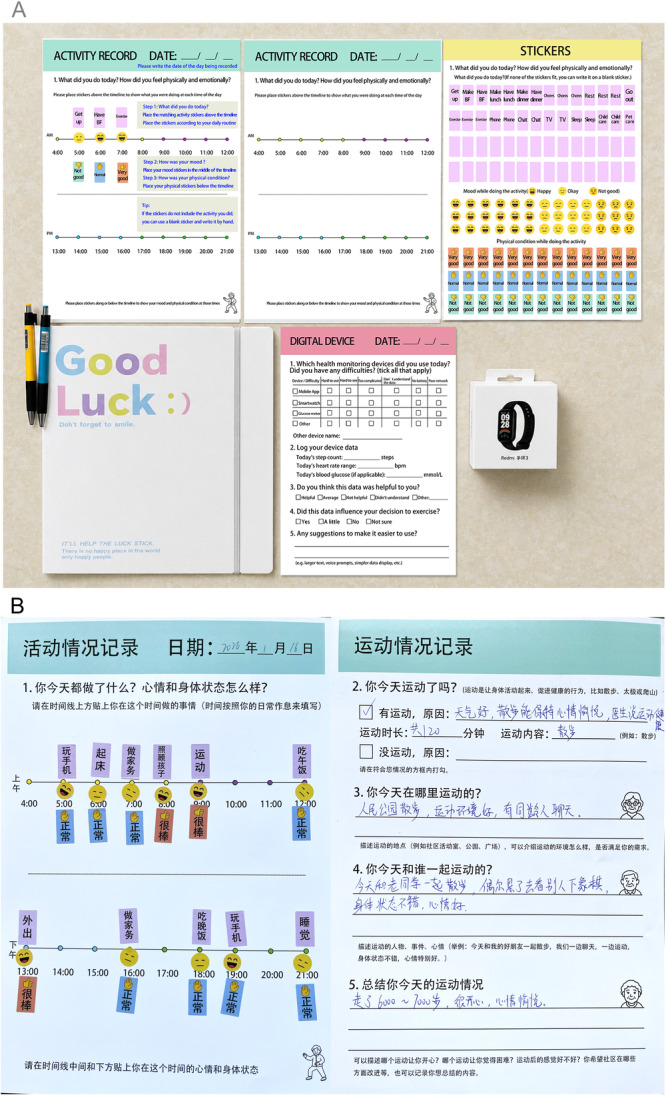
(A) The full paper‐based design probe toolkit, including activity and exercise record sheets, a sticker sheet, a digital device record sheet and a wearable device used to support the recording of health‐related digital data. (B) Examples of completed daily activity and exercise record sheets.

Design probes were introduced to generate user information, support participation and build dialogue between participants and researchers, while also making everyday experiences visible over time [55]. In this study, the probes were used to capture routines, emotions, social interactions and experiences with health‐related technologies in daily life that might not be fully revealed through one‐off interviews [37]. Eight older adults with T2DM took part in a 2‐week probe study. The paper‐based toolkit included structured diaries, visual markers, timelines and simple prompts. The materials were designed using age‐friendly principles, with an emphasis on visual expression, low cognitive burden and flexible participation [38, 39, 56]. The probe records captured contextual data on recurring behaviours, needs and emotional experiences, which then informed the clarification interviews. Figure 1 shows the design probe toolkit and examples of completed record cards from one older adult participant with T2DM.

#### 2.4.3 Stage 3: Clarification Interviews

After the probe stage, clarification interviews were conducted to address the fragmented nature of probe materials, reduce possible researcher misinterpretation and support later discussion of service ideas [39, 55]. These interviews focused on participants' interpretations of their exercise records, emotional responses and preferences for feedback and data sharing. Follow‐up clarification interviews were planned with the eight older adults with T2DM who completed the probe stage; one later withdrew because of time and health constraints, and the interviews were completed with seven participants. Each interview lasted approximately 30–60 min and was audio‐recorded. This stage helped refine interpretation of the probe materials and generated more detailed insights to support the subsequent co‐design workshop.

#### 2.4.4 Stage 4: Co‐Design Workshop

**Figure 2:**
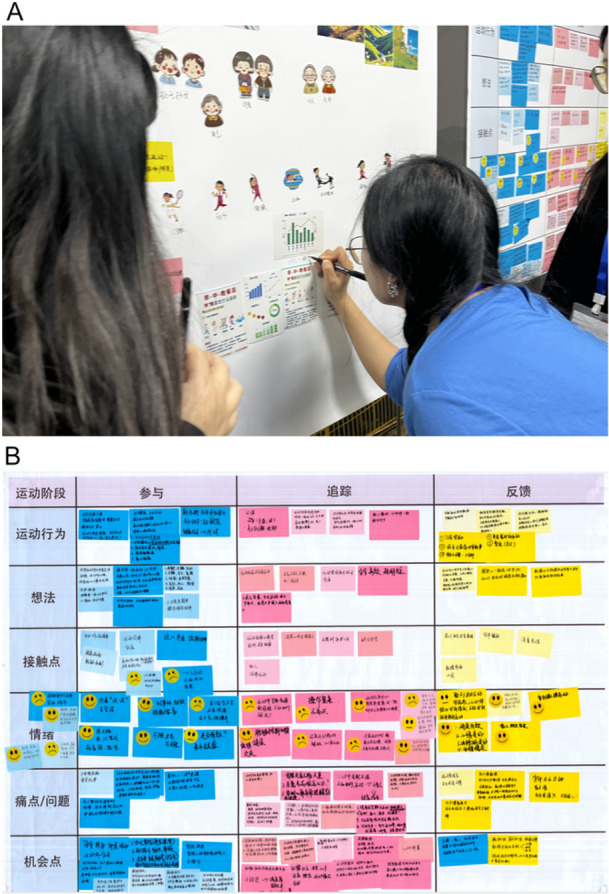
(A) Participants from different stakeholder groups discussing ideas during the co‐design workshop. (B) An example of a user journey map developed during the workshop.

The co‐design workshop brought together multiple stakeholders to contribute their experiences and expertise to the development of service ideas, helping ensure that proposed solutions were grounded in lived experience and practical knowledge [16, 22]. The workshop was designed to translating earlier findings into shared discussion and design exploration around XR‐supported exercise services. It involved eight participants: three older adults with T2DM, two community workers, one healthcare professional and two XR designers. The workshop lasted approximately 3 hours and was video‐recorded. Video excerpts from earlier interviews were used as trigger materials to support shared understanding and stimulate discussion, drawing on EBCD‐inspired approaches [57]. Participants then took part in group discussion, user journey mapping and scenario‐based idea generation. Participation was supported through age‐friendly facilitation strategies, including pre‐workshop orientation and visual and audio support [58, 59]. The workshop produced shared insights, user journey maps and early service ideas, which were then taken forward into the evaluation interviews. Figure 2 shows examples of the co‐design workshop process and outputs.

#### 2.4.5 Stage 5: Evaluation Interviews

Evaluation interviews were used to examine participants' understanding and acceptance of the proposed service model after they had engaged with concrete design ideas and workshop outputs [60]. The interviews explored responses to the proposed XR‐based exercise service model, including its service process, engagement mechanisms, tracking mechanisms and feedback arrangements. Follow‐up interviews were conducted with participants who had taken part in the co‐design workshop. Each interview lasted approximately 30–60 min and was audio‐recorded. This stage provided qualitative feedback on the clarity, perceived usefulness and relevance of the proposed concepts and informed further refinement of both the service ideas and the methodological process. The stage‐by‐stage research process, including participant numbers at each stage, is summarised in Table 1.

**Table 1:** Research process across the five study stages.

Stage	Method	Participants	Duration	Recording
Stage 1	Exploratory semi‐structured interviews	20 participants: 8 older adults with T2DM, 6 community workers, 4 healthcare professionals, 2 XR designers	30–60 min	Audio recording
Stage 2	Design probes	8 participants: 8 older adults with T2DM	2 weeks	Paper‐based recording
Stage 3	Clarification interviews	7 participants: 7 older adults with T2DM	30–60 min	Audio recording
Stage 4	Co‐design workshop	8 participants: 3 older adults with T2DM, 2 community workers, 1 healthcare professional, 2 XR designers	3 hours	Audio/video recording
Stage 5	Evaluation interviews	8 participants: 3 older adults with T2DM, 2 community workers, 1 healthcare professional, 2 XR designers	30–60 min	Audio recording

### 2.5 Data Analysis

All qualitative data were analysed using Braun and Clarke's six‐phase thematic analysis framework [61], including familiarisation, initial coding, theme development, theme refinement, theme definition and reporting. This approach was appropriate for identifying patterns across the different stages of the co‐design process while remaining sensitive to participants' lived experiences.

The main analytic units were text‐based materials derived from interviews, design probe records and co‐design workshop outputs. Interview and workshop discussions were audio‐recorded and transcribed, while design probe content and written or drawn workshop outputs were converted into editable text for analysis. Workshop activities were also video‐recorded to support interpretation of group interaction and collaborative processes. Important segments were highlighted during data preparation to support familiarisation and coding.

Analysis was conducted in NVivo (version 15). Coding began with the exploratory interview transcripts, using an open coding approach to identify key experiences, concerns and suggestions. The codes were then grouped into broader categories and refined into themes. The analysis was guided by three aims: to address the research questions and objectives, to examine and refine the proposed framework and to remain open to new findings emerging from the data.

The analysis involved both within‐stage interpretation and cross‐stage integration. Earlier‐stage data informed the design and interpretation of later stages, while clarification and evaluation interviews were used to confirm, extend and refine the understanding developed from earlier materials. In addition to NVivo‐based coding, Miro was used as a visual synthesis tool to organise coded content across stages and stakeholder groups. It supported concept mapping and journey‐based visual structuring to aid comparison, clustering and shared understanding of the data, particularly among older adults and other stakeholders.

Methodological rigour was supported through triangulation across methods and participant groups [62, 63]. Semi‐structured interviews, design probes, clarification interviews, co‐design workshop activities and evaluation interviews provided complementary forms of qualitative data across the five stages of the study [64, 65]. The inclusion of older adults with T2DM, community workers, healthcare professionals and XR designers enabled comparison across stakeholder perspectives and supported interpretation across data sources of the methodological findings. Clarification and evaluation interviews further helped refine and extend interpretations developed from earlier stages. These strategies contributed to a more credible and transparent interpretation of the methodological findings [66, 67]. The workflow of the five‐stage co‐design methodology and its associated data analysis processes is summarised in Table 2.

**Table 2:** Workflow of the five‐stage co‐design methodology and data analysis.

Stage	Data collection	Analytic contribution
Stage 1 exploratory interviews	Semi‐structured interviews Audio recordings Interview transcripts	Identify initial experiences, needs, barriers and facilitators Inform the design of the probe toolkit
Stage 2 design probes	Paper‐based probe materials Daily logs Emotional records Visual and written entries	Capture everyday routines, behaviours and emotions in context Extend understanding beyond one‐off interviews
Stage 3 clarification interviews	Follow‐up interviews Audio recordings Interview transcripts	Clarify and confirm the meanings of probe entries Refine interpretation of probe materials
Stage 4 co‐design workshop	Group discussions Video recordings Workshop transcripts User journey maps Service concept sketches	Translate earlier insights into shared discussion and service ideas Generate collaborative design ideas and methodological feedback
Stage 5 evaluation interviews	Follow‐up evaluation interviews Audio recordings Interview transcripts	Gather feedback on later‐stage design ideas Assess clarity, acceptability and relevance of proposed concepts Inform further refinement of the methodology and service model
**Data analysis:** Transcription and data preparation → open coding in NVivo → category formation → theme development and refinement → cross‐stage integration → visual synthesis in Miro → interpretation and reporting.		

## 3 Results

### 3.1 Participant Characteristics

The full study sample comprised 24 participants across four stakeholder groups. Participants varied in age, gender and educational background, and their stage‐specific participation reflected the sequential design of the study. Detailed participant characteristics are presented in Table 3.

**Table 3:** Participant characteristics.

Participant group	ID	Age	Gender	Education (degree)	Stage(s) participation
Older adults with T2DM	P01	70	M^a^	Secondary	Stages 1–3
Older adults with T2DM	P02	61	F^b^	Doctorate	Stages 1–5
Older adults with T2DM	P03	60	M	Secondary	Stages 1–3
Older adults with T2DM	P04	60	M	Secondary	Stages 1–3
Older adults with T2DM	P05	71	F	Secondary	Stages 1–3
Older adults with T2DM	P06	61	M	Secondary	Stages 1–3
Older adults with T2DM	P07	78	M	Secondary	Stages 1–3
Older adults with T2DM	P08	72	F	Primary	Stages 1, 2
Older adults with T2DM	P09	60	F	Secondary	Stages 4, 5
Older adults with T2DM	P10	77	M	Bachelor	Stages 4, 5
Community workers	CW01	42	F	Not reported	Stage 1
Community workers	CW02	36	F	Not reported	Stage 1
Community workers	CW03	40	F	Not reported	Stage 1
Community workers	CW04	41	M	Not reported	Stage 1
Community workers	CW05	23	F	Not reported	Stage 1
Community workers	CW06	23	F	Not reported	Stages 1, 4, 5
Community workers	CW07	30	F	Not reported	Stages 4, 5
Healthcare professionals	HP01	30	F	Master	Stage 1
Healthcare professionals	HP02	29	F	Master	Stage 1
Healthcare professionals	HP03	32	F	Master	Stage 1
Healthcare professionals	HP04	44	M	Master	Stage 1
Healthcare professionals	HP05	31	F	Master	Stages 4, 5
Designers	D01	38	F	Master	Stages 1, 4, 5
Designers	D02	30	F	Master	Stages 1, 4, 5

^a^ M: male.

^b^ F: female.

### 3.2 Acceptability

Participants had positive experiences of taking part in the research and expressed willingness to engage in the interviews, design probe activities and co‐design workshops. Three themes were identified: low perceived burden and ease of participation, positive emotional engagement with the research tools and willingness to continue participating.

#### 3.2.1 Low Perceived Burden and Ease of Participation

Participants generally described the research activities as manageable and easy to follow. Older adults reported that the interviews were closely related to daily life and were easy to understand and answer. One participant commented that the interview topics were ‘very close to daily life, easy to understand, and the timing and rhythm of the interview were relaxed’ (OA04). The design probe tasks were also described as simple and low pressure, and the time required to complete them was generally considered acceptable. One participant noted that the task was ‘easy’ (OA01), while another said, ‘No pressure. That is fine. If there are stickers, I use the stickers’ (OA04). Similarly, workshop participants described the pacing and structure of the activities as comfortable and manageable, explaining that ‘the pacing felt comfortable and the questions progressed step by step in a way that matched our thinking process’ (CW06).

#### 3.2.2 Positive Emotional Engagement With the Research Tools

In addition to being easy to complete, the probe activities also generated positive emotional responses. In particular, stickers and other visual elements were seen as engaging and enjoyable. One participant described the experience as relaxed and playful: ‘It is more relaxed. It is like playing in kindergarten, because I have not played with stickers for a long time. It is quite okay, and easier to understand and express. Your stickers are fine, pretty good’ (OA04). Familiar and simple visual elements appeared to reduce formality and support a more positive participation experience.

#### 3.2.3 Willingness to Continue Participating

Participants expressed a clear willingness to continue participating in later stages of the study. One participant stated, ‘I would be happy to try the design later and give more feedback’ (OA01). Active engagement during the co‐design workshop further reflected participants' interest in the process, and some participants also described the probe activities as personally valuable. For example, one participant explained, ‘When I look back at the records every day, I know what I did, and I can place some self‐discipline on myself’ (OA06). These responses show that participants not only accepted the research methods but also found value in the process.

### 3.3 Appropriateness

The research methods were perceived as appropriate for older adults, although some aspects of the materials and activity formats required clarification or minor adjustment. Two themes were identified: the general comprehensibility of the research materials and the importance of age‐friendly communication formats.

#### 3.3.1 General Comprehensibility of Research Materials

The research materials were largely seen as easy to understand. During the design probe stage, several older adults noted that the instructions and recording tasks were clear and closely related to everyday experience. For example, one participant said that ‘It was very easy to understand. They are all common‐sense things from daily life. There was nothing hard to understand’ (OA04). A small number of participants also reported that some items required additional clarification, particularly numerical indicators such as heart rate and blood glucose values. One older adult noted that ‘Some of the numerical values were unclear, especially the heart rate and blood glucose values. I asked you about them before I filled them in. The rest were about daily life’ (OA06).

Feedback from the co‐design workshop similarly suggested that many discussion topics were understandable, especially when participants could draw on their own experiences. One community worker reported that ‘The first part of the workshop discussion was easy to engage with because everyone could simply draw on their own experiences’ (CW06). One older adult described the proposed exercise design as ‘quite easy to understand’ and ‘very straightforward’ (OA10).

#### 3.3.2 Importance of Age‐Friendly Communication Formats

Participants frequently highlighted the importance of visual and age‐friendly communication formats. Many older adults reported that stickers and icons made the recording process easier, more intuitive and less dependent on writing. One participant pointed out that stickers were ‘more practical and clearer’, adding that ‘with stickers it is easier to express things clearly’ (OA01). Others also described stickers as easier to understand and use than handwriting (OA02, OA04, OA06).

Visual communication was considered helpful for age‐related changes in vision. One participant explained, ‘Since I have presbyopia now, using icons works better and is more convenient’ (OA07). One participant also mentioned that ‘In daily life, typing is more convenient for me, and handwriting is not very convenient. If there were an electronic or digital version, it would be easier for me because I could type. Writing by hand is a bit troublesome’ (OA02). Participants in the co‐design workshop indicated that images helped them understand the exercise design concepts more easily (OA10).

### 3.4 Feasibility

Participants' responses revealed several practical considerations affecting the feasibility of implementing the proposed approach in community settings. Two themes were identified: resource and organisational requirements, and practical participation constraints.

#### 3.4.1 Resource and Organisational Requirements

Participants emphasised that implementing XR‐based exercise activities in community settings would require substantial organisational support, particularly in funding, physical space, equipment and staff. Community workers noted that staff would be needed both to help older adults use the equipment and to manage devices during activities. As one participant explained, ‘You need someone to help them wear the equipment, and you also need someone to manage the devices’ (CW01). The same participant also noted the need for appropriate space, stating, ‘Not every community has such a good environment’ (CW01).

Funding was repeatedly identified as a major condition for implementation. One community worker stated directly that ‘Funding is definitely the most important thing’ (CW04), while others highlighted the need for coordinated support involving venue, personnel, equipment management and ongoing maintenance (CW02; CW03). These responses indicate that implementing XR‐based exercise programmes in community settings would require institutional support in staffing, space, funding and organisational coordination.

#### 3.4.2 Practical Participation Constraints

Participants identified practical constraints affecting continued involvement, including health conditions, scheduling conflicts and fatigue. During the follow‐up stage after the design probe period, one older adult withdrew because of health issues and time constraints. Some participants reported that the co‐design workshop could feel tiring when sessions were too long. As one participant noted, ‘It is a bit tiring because the time is too long’ (OA09). These findings suggest that although the research methods were feasible, participation could still be shaped by practical factors such as health, time availability and the length of participatory activities.

### 3.5 Methodological Refinement During Implementation

Beyond evaluating acceptability, appropriateness and feasibility, the study also generated methodological insights during implementation. Participants' feedback and researcher reflections informed several refinements to the research process. Two themes were identified: participant‐informed methodological improvements and researcher‐initiated methodological adjustments.

#### 3.5.1 Participant‐Informed Methodological Improvements

Participants' feedback during the design probe and co‐design workshop stages directly informed refinements to the visual design of research materials, the clarity of workshop communication and the format and duration of activities.

First, several participants suggested improving the readability and visual clarity of the probe materials. One participant recommended larger fonts and more visual presentation, noting that ‘I hope there could be more visual text, and the font should be bigger because sometimes people cannot see clearly without glasses’ (OA02). Participants also highlighted the need to refine some stickers and icons, especially where meanings were unclear or emotionally inappropriate. For example, one participant commented that the thumbs‐down icon for discomfort felt too negative and should be changed (OA02). Others found the ‘okay’ sticker unclear or suggested that a smiling symbol would be easier to understand (OA07).

Second, participants reported difficulty understanding some workshop concepts, particularly user journey mapping. A community worker noted that ‘The user journey mapping was a bit difficult to understand because there was no specific explanation of the design concept’ (CW06). Others said that the terminology should be simplified and explained more clearly in advance. A healthcare professional also suggested that ‘The process could be simplified, and it might help to create a short video or provide similar examples’ (HP05).

Finally, participants suggested that some aspects of the activity format and duration could be adjusted. For example, one participant noted that the daily probe recordings felt slightly long and could be shorter (OA05). Another suggested that recording exercise duration in hours rather than minutes would be easier for longer activities (OA04). Participants also indicated that the overall workshop length could be demanding for some older adults. One participant explained that ‘The total time should be a bit shorter, because older people may not be able to sit for several hours’ (OA10).

#### 3.5.2 Researcher‐Initiated Methodological Adjustments

In addition to participant‐driven feedback, the research team also made several methodological adjustments during implementation. One adjustment was the introduction of a pre‐training session before the co‐design workshop to explain the workshop structure, key concepts and the use of materials. This aimed to reduce participation barriers and support more confident engagement in workshop discussions [68].

Another adjustment concerned the presentation of background information. Written summaries of earlier interview findings were initially planned for use before the workshop, but these were replaced with video‐based trigger materials because some older adults might find long text difficult to read [69]. Short video clips from the interviews helped participants understand others' experiences and perspectives more easily and engage more actively in the subsequent co‐design discussions.

Furthermore, the research team refined the semi‐structured interview guide during the research process. Some interview questions that were less directly related to the research objectives were removed to make the interview process more focused [70]. This adjustment helped reduce the time burden on participants and improved the overall efficiency of the interviews while maintaining the relevance of the collected data.

These adjustments show how the methodology was refined during the study in response to both participant feedback and practical considerations encountered during implementation.

## 4 Discussion

### 4.1 Methodological Insights From Implementing Co‐Design Research With Older Adults

The findings of this study suggest that effective co‐design with older adults in community health contexts depends not only on providing opportunities for participation but also on how methods are structured, explained and experienced across stages. Three methodological insights were especially important in this regard. These related to the accessibility of design‐related concepts, the refinement of visual communication tools in relation to older adults' interpretive preferences and the need to balance workshop duration with participant comfort and depth of discussion. These insights point to a key methodological contribution of this study, namely that co‐design with older adults may require adaptation at three interconnected levels: conceptual, visual and procedural.

One key methodological insight concerns the conceptual accessibility in co‐design with older adults. Although methods such as user journey mapping are widely used in design research, some participants found the related terminology and conceptual structures difficult to understand. This suggests that accessibility in co‐design is not limited to physical or sensory access, but also depends on whether design concepts are communicated in ways that are meaningful within participants' everyday communicative practices [71]. Previous studies similarly note that professional and disciplinary specific language can be exclusionary [72] and may limit participation when methods are not sufficiently adapted to participants' backgrounds and communication styles [71, 73]. In addition, work on co‐design with older adults has highlighted the importance of preparatory work, expectation‐setting, trust‐building and tailored facilitation in enabling inclusive participation, particularly when participants are asked to engage with unfamiliar design or digital processes [26, 58]. From this perspective, conceptual accessibility is not simply a matter of clearer explanation, but is closely related to how co‐design activities are introduced, structured and facilitated. The present findings extend these discussions by showing that difficulties with terminology and conceptual structure may persist even when participants are willing to take part, and that clearer preparation and more structured facilitation are especially important when unfamiliar co‐design concepts or tools are involved [74]. Introductory explanations, concrete examples and simple instructional resources may therefore help older adults become more familiar with co‐design activities and participate with greater confidence [58].

A second methodological insight relates to the interpretive limits of visual communication tools. Although icons and stickers reduced reliance on written text and supported participation, some participants found certain symbols difficult to interpret or emotionally inappropriate. This highlights that visual tools in co‐design are not inherently inclusive. Their value lies in whether they are recognisable, easy to interpret and emotionally acceptable to older participants. Recognition of graphical symbols depends on prior knowledge and memory [75], and older adults tend to benefit more from concrete and familiar icon styles than from abstract graphics [76, 77]. At the same time, visual formats are not necessarily more acceptable than text‐based ones in all cases. Some evidence suggests that older adults may respond more positively to text interfaces than younger users, and may show better comprehension and higher usability ratings when text‐based formats are familiar to them [78]. This indicates that no single format can be assumed to be universally inclusive for older adults. The present findings are consistent with this broader literature, but they also extend it by showing that emotional appropriateness is an additional consideration when visual tools are used in participatory research with older adults [78]. Recent design guidance further suggests that visual symbols may work better when combined with textual cues [79] and refined through participatory testing with older users [80]. These findings reinforce the importance of iterative testing and refinement of visual tools with older adults to improve clarity, inclusiveness and emotional acceptability [81].

A third methodological insight highlights the need to treat session duration as a methodological design issue rather than a purely logistical one. Although participants generally described the workshop rhythm as manageable, longer sessions could still lead to fatigue in the later stages. This aligns with previous studies showing that extended activities may place greater demands on older adults because of age‐related sensory, cognitive and communication changes [82]. However, other work and the present findings also suggest that sessions shortened too far may reduce the depth of discussion and limit the reflective and collaborative contribution that co‐design requires [83]. These findings indicate that the relationship between accessibility and discussion quality is not straightforward. Rather than simply reducing duration, co‐design sessions with older adults may need to balance manageable pacing, clearer task segmentation and built‐in opportunities for rest with sufficient time for collaborative reflection and idea generation [84, 85].

These insights show that the proposed framework offers methodological value not simply because it enabled participation, but because it clarifies how co‐design with older adults can be made more conceptually accessible, visually understandable and procedurally manageable in community health research.

### 4.2 Practical Methodological Principles for Future Co‐Design Research

Despite the conceptual, visual and procedural challenges identified in the study, the study also suggests a set of practical methodological principles that may inform future co‐design research and design practice with older adults.

#### 4.2.1 Begin With Individual Experience Before Collective Co‐Design

One practical methodological principle is to begin with individual experience before moving into collective co‐design. Exploratory interviews and design probes helped ground later workshop discussions in participants' everyday realities rather than abstract assumptions. This is consistent with EBCD approaches, which typically begin with observation and interviews before progressing to joint workshops and co‐design groups [86]. The present findings further show that this sequencing is especially important when working with older adults, because it allows later collective discussion to build on familiar lived experience rather than requiring participants to engage immediately with abstract design ideas.

#### 4.2.2 Use a Staged and Progressive Process

A second principle is to structure co‐design as a staged and progressive process. The multi‐stage design enabled participants to engage gradually and reduced the pressure of being introduced to unfamiliar activities all at once. Healthcare co‐design frameworks likewise distinguish pre‐design, co‐design and post‐design phases in order to support more systematic development and participation [87]. For older adults with T2DM, this kind of sequencing may be particularly helpful because it creates a more gradual pathway into participation and allows familiarity and confidence to build over time.

#### 4.2.3 Combine Interviews, Design Probes and Clarification Interviews

A third principle is to combine interviews, design probes and clarification interviews in a complementary sequence. This combination made participants' routines, emotions and behavioural changes more visible and helped researchers interpret participant‐generated materials more effectively. Interviews enabled participants to share personal views and difficulties, particularly in relation to exercise and technology use [88]. Design probes then allowed participants to record and reflect on their own everyday experiences over time, providing participant‐generated accounts rather than relying only on researcher‐led questioning. Follow‐up clarification interviews further helped researchers and participants revisit these records and discuss them in more natural and in‐depth ways [39].

#### 4.2.4 Use Age‐Friendly, Low‐Burden Materials

A fourth principle is to use age‐friendly, low‐burden materials throughout the co‐design process. Diaries, icons, stickers and simple prompts reduced reliance on long written responses and supported more flexible forms of expression. Previous research has similarly emphasised large‐print materials, optional participation, caregiver support and low‐pressure probe tasks for older adults [38, 39]. Materials of this kind can also shape how older adults express their experiences and remain engaged in the research process.

#### 4.2.5 Provide Preparatory Support Before Workshops

A fifth principle is to provide preparatory support before workshops. Explanations, examples and visual materials helped participants understand the process, reduced uncertainty and improved confidence before the co‐design sessions began. Similar work has highlighted the value of demonstrations, videos and pre‐training in preparing older adults for participation [58, 68].

#### 4.2.6 Break Workshop Activities Into Manageable Tasks and Involve Multiple Stakeholders

A sixth principle is to structure workshop activities into manageable tasks while involving multiple stakeholder groups. This helped reduce cognitive and physical burden for older participants and supported participation through a more gradual and structured workshop process. The involvement of multiple stakeholders also broadened discussion by bringing together experiential, organisational, clinical and design perspectives. Previous studies similarly emphasise the need for careful pacing, breaks and structured planning in participatory workshops [74, 89].

#### 4.2.7 Include a Final Evaluation Stage

A final principle is to include a final evaluation stage after collective co‐design activities. Evaluation interviews helped assess whether the proposed service model was clear, acceptable and relevant after the workshop. Previous studies have also used reflective evaluation interviews after co‐design activities to examine how participants understand and respond to proposed ideas [60].

### 4.3 Limitations of the Study

Several limitations should be noted. First, the study involved a relatively small number of participants, which may limit the generalisability of the findings. Second, the multi‐stage co‐design process required considerable time, coordination and facilitation, meaning that implementation may depend on funding, staff support and appropriate community spaces [73]. Third, some co‐design activities may still place cognitive and interpretive demands on older participants, particularly when unfamiliar concepts or tools are introduced [71]. Fourth, the study did not include quantitative measures of acceptability, appropriateness and feasibility. Future research may benefit from incorporating validated implementation measures to complement the qualitative findings and strengthen evaluation [90]. These limitations suggest that while the approach offers value for engaging older adults in health research, its implementation requires careful planning and sufficient resources.

### 4.4 Implications for Future Research and Practice

The findings suggest that the co‐design approach developed in this study has relevance beyond exercise and community‐based activities for older adults with T2DM. It may also be applicable in other long‐term condition contexts that require sustained self‐management and support for health behaviour. Although the approach was developed in relation to a technology‐supported intervention, its implications extend more broadly to the design and improvement of community health services and health promotion programmes [21]. The study also highlights the value of developing more structured co‐design resources, such as toolkits or practical guidance [19], to support accessible and inclusive research with older adults.

## 5 Conclusion

This study developed and examined an age‐adapted co‐design methodology for community health research involving older adults with T2DM. The findings suggest that a staged process grounded in lived experience and supported by age‐friendly, low‐burden materials can enable participation in ways that are acceptable, appropriate and feasible. Beyond demonstrating the practical value of the framework, the study contributes methodological insight into how co‐design with older adults can be made more conceptually accessible, visually understandable and procedurally manageable. The proposed methodology offers a structured and practical approach to involving older adults in community health research and provides a useful basis for future co‐design research in similar contexts.

## Author Contributions

**Jiangpan Niu:** conceptualization, methodology, software, data curation, resources, formal analysis, project administration, validation, visualization, writing – review and editing, writing – original draft, investigation. **Yuanyuan Yin:** conceptualization, methodology, data curation, supervision, writing – review and editing, validation, formal analysis, visualization, investigation. **Shan Wang:** conceptualization, methodology, data curation, validation, formal analysis, supervision, writing – review and editing, visualization, investigation.

## Funding

The authors have nothing to report.

## Ethics Statement

This study was approved by the University of Southampton Research Ethics Committee, Southampton, UK (109247). Written and verbal informed consent was obtained from all participants.

## Conflicts of Interest

The authors declare no conflicts of interest.

## Supporting information

Supporting File 1

Supporting File 2

## Data Availability

The data that support the findings of this study are available on request from the corresponding author. The data are not publicly available due to privacy or ethical restrictions.
