# Supplementary material for: An Age‐Adapted Co‐Design Methodology for Community Health Research Involving Older Adults With Type 2 Diabetes
Source: Health Expect. 2026 Jun 7;29(3):e70718. doi: 10.1111/hex.70718 (PMC13243777; doi:10.1111/hex.70718)
Supplement: Supplementary file 1 — Supporting File 1 [file HEX-29-e70718-s002.pdf]

Appendix A: Sample Design Probe Toolkit

Instructions for Completing the Design Probe Cards

This booklet is designed to understand your daily activities, exercise, and use of digital devices. There are no right or wrong answers. This is not a test or a task.

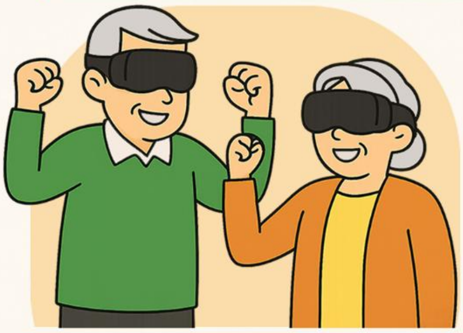

Over 10–14 days

Please complete the cards based on your real situation by placing stickers, writing short notes, ticking boxes, or making simple marks.

1 What to complete

- 7 days of activity/exercise record cards (Pages 1–7)
- 3 days of digital device record cards (Page 8)

2 How to use Pages 1–7

- Choose any 7 days within the 10–14 days
- The 7 days do not need to be consecutive
- You can complete them using stickers and handwriting

3 How to use Page 8

- Choose any 3 days within the 10–14 days
- The 3 days do not need to be consecutive

4 Please remember

- You do not need to fill it in every day
- If you do not want to fill it in one day, that is completely fine
- If you forget, you can complete it the next day
- It is fine to write more or less

If you have any questions while filling it in, please feel free to contact me.

ACTIVITY RECORD DATE: ____/ ___/ ___

1. What did you do today? How did you feel physically and emotionally?

Please place stickers above the timeline to show what you were doing at each time of the day

AM

4:00 5:00 6:00 7:00 8:00 9:00 10:00 11:00 12:00

PM

13:00 14:00 15:00 16:00 17:00 18:00 19:00 20:00 21:00

Please place stickers along or below the timeline to show your mood and physical condition at those times

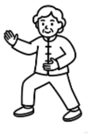

ACTIVITY RECORD DATE: ____/ ___/ ___

Please write the date of the day being recorded

1. What did you do today? How did you feel physically and emotionally?

Please place stickers above the timeline to show what you were doing at each time of the day

Get up

Have BF

Exercise

Step 1: What did you do today?

Place the matching activity stickers above the timeline

Place the stickers according to your daily routine

AM

4:00 5:00 6:00 7:00 8:00 9:00 10:00 11:00 12:00

Not good

Normal

Very good

Step 2: How was your mood ?

Place your mood stickers in the middle of the timeline

Step 3: How was your physical condition?

Place your physical stickers below the timeline

Tip:

If the stickers do not include the activity you did, you can use a blank sticker and write it by hand.

PM

13:00 14:00 15:00 16:00 17:00 18:00 19:00 20:00 21:00

Please place stickers along or below the timeline to show your mood and physical condition at those times

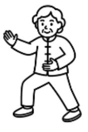

EXERCISE RECORD

2. Did you exercise today?

(Exercise means getting your body moving to improve health, for example walking, tai chi, or climbing stairs.)

☐ Yes, reason: ____________________________________

Duration: __________minutes Type of exercise: __________

☐ No, reason: ____________________________________

Please tick the box that applies to you.

3. Where did you exercise today?

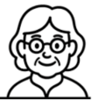

____

____

Describe where you exercised (for example, a community activity room, park, or square). You can also describe what the exercise environment was like and whether it met your needs.

4. Who did you exercise with today?

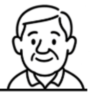

____

____

____

Describe who you exercised with, what happened, and how you felt (for example: Today I went for a walk with my good friend. We chatted while walking. My physical condition was good, and I felt very happy.)

5. Summary of your exercise today

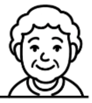

____

____

____

You can also describe which exercise made you happy, which exercise felt difficult, how you felt after exercising, and what improvements you would like the community to make. You may also record any other thoughts you want to summarise.

STICKERS

1. What did you do today? How did you feel physically and emotionally?

What did you do today?(If none of the stickers fit, you can write it on a blank sticker.)

Get up	Make BF	Have BF	Make lunch	Have lunch	Make dinner	Have dinner	Chores	Chores	Chores	Rest	Rest	Rest	Go out
Exercise	Exercise	Exercise	Phone	Phone	Chat	Chat	TV	TV	Sleep	Sleep	Child care	Child care	Pet care

Mood while doing the activity(Happy Okay Not good)

Physical condition while doing the activity

Very good	Very good	Very good	Very good	Very good	Very good	Very good	Very good	Very good	Very good	Very good	Very good	Very good
Normal	Normal	Normal	Normal	Normal	Normal	Normal	Normal	Normal	Normal	Normal	Normal	Normal
Not good	Not good	Not good	Not good	Not good	Not good	Not good	Not good	Not good	Not good	Not good	Not good	Not good

DIGITAL DEVICE DATE: ___/ __/ __

1. Which health monitoring devices did you use today? Did you have any difficulties? (tick all that apply)

Device / Difficulty	Hard to use	Hard to see	Too complicated	Don’t understand the data	No battery	Poor network
☐ Mobile App	☐	☐	☐	☐	☐	☐
☐ Smartwatch	☐	☐	☐	☐	☐	☐
☐ Glucose meter	☐	☐	☐	☐	☐	☐
☐ Other	☐	☐	☐	☐	☐	☐

Other device name: ______________________

2. Log your device data

Today‘s step count: ______________ steps

Today‘s heart rate range: ______________ bpm

Today‘s blood glucose (if applicable): ______________ mmol/L

3. Do you think this data was helpful to you?

☐ Helpful ☐ Average ☐ Not helpful ☐ Didn‘t understand ☐ Other:________

4. Did this data influence your decision to exercise?

☐ Yes ☐ A little ☐ No ☐ Not sure

5. Any suggestions to make it easier to use?

____

____

(e.g. larger text, voice prompts, simpler data display, etc.)
