# Supplementary material for: An Age‐Adapted Co‐Design Methodology for Community Health Research Involving Older Adults With Type 2 Diabetes
Source: Health Expect. 2026 Jun 7;29(3):e70718. doi: 10.1111/hex.70718 (PMC13243777; doi:10.1111/hex.70718)
Supplement: Supplementary file 2 — Supporting File 2 [file HEX-29-e70718-s001.pdf]

Appendix B: Sample Co-Design Workshop Materials and Process

Co-Design Workshop Guide

Work together with stakeholders to explore the future of XR exercise services. This workshop brings together researchers, older adults with T2DM, designers, healthcare professionals, and community workers to understand the needs of older adults with T2DM and the community context, map the XR exercise journey, and co-create ideal XR exercise scenarios.

Workshop Agenda

1. Welcome and introduction

Build trust and explain the co-design goals and tools.

2. Focus group discussion

Discuss the three key mechanisms: engagement, tracking, and feedback.

3. User journey map co-creation

Work together to map the full XR exercise journey and key turning points.

4. XR co-design

Use visual prompts and creative collage to express ideal exercise scenarios.

Part 1: Welcome and Introduction

Goal: To establish a safe and open environment and review earlier research findings.

Steps:

1. Welcome and self-introductions (researchers and participants);
2. Brief explanation of workshop objectives and agenda;
3. Clarify the value of co-design: participants are "co-creators";
4. View anonymised video excerpts from earlier interviews with different stakeholder groups to stimulate discussion;

5. Introduce tools (e.g., UJM templates, emotion stickers, data visuals) and usage rules for accessibility across diverse educational backgrounds.

Introduce user journey mapping

Exercise Stage	Engagement	Tracking	Feedback
Exercise Behaviour			
Thoughts			
Touchpoints			
Emotions			
Pain Points / Challenges			
Opportunities			

1. Exercise Behaviour:

What did the user do at this stage?

Record the specific actions that took place at this point in the journey, such as starting exercise, receiving a recommendation, checking data, or receiving a reward.

Simple example:

The participant put on the headset, chose a walking game, and started exercising.

2. Thoughts

What was the user thinking at this stage?

Note the user's immediate thoughts, concerns, expectations, or preferences. This helps us understand how they interpreted the experience.

Simple example:

"I hope this will be easy to use."

"I'm not sure whether I can do this by myself."

3. **Touchpoints**

What did the user interact with at this stage?

Identify the people, tools, services, or system features the user came into contact with during the experience.

Simple example:

The participant spoke to a community worker, used the VR headset, and looked at the progress screen.

4. **Emotions**

How did the user feel at this stage?

Show the user's emotional response at different points in the journey. You can use emoji stickers or colours to show positive, neutral, or negative feelings.

Simple example:

The participant felt curious at the beginning, frustrated when something did not work, and happy after finishing the exercise.

5. **Pain Points/ Challenges**

Where did difficulties or negative experiences happen?

Identify the moments where the user faced barriers, confusion, discomfort, or other problems that affected the experience.

Simple example:

The text on the screen was too small to read.

The participant did not know what to do next.

6. **Opportunities**

Where could the experience be improved?

Look at the pain points and identify possible ways to improve the service, system, or support.

Simple example:

Add larger text, clearer instructions, or voice guidance to help the participant continue more confidently.

Part 2: Focus Group Discussion

Goal: Explore participants' expectations and interpretations of the ETF mechanisms through guided questions about how different stakeholders understand these service mechanisms and what they expect from them.

Discussion Themes:

1. **Engagement**
   Key question: What do you need most when starting exercise?
   Explore users' motivation, barriers, and support needs.
2. **Tracking**
   Key question: What behaviours and data would you be willing to have tracked?
   Explore privacy boundaries and acceptance of data collection.
3. **Feedback**
   Key question: How would you like to see your progress? Who should the feedback be shared with?
   Explore preferred feedback formats and willingness to share.

Facilitation tip:

Encourage participants to share concrete examples and personal experiences. Record key insights and emotional responses for the next stage.

Sharing Focus Group Findings:

- Summarise key insights from different perspectives
- Highlight shared and individual user needs
- Identify potential design opportunities
- Describe specific situations that created resonance

Facilitators support cross-disciplinary dialogue and help participants recognise different viewpoints, laying the groundwork for journey map co-creation.

Focus group discussion materials

What do you need most when starting exercise?

What behaviours and data would you be willing to have tracked?

How would you like to see your progress? Who should receive the feedback data?

Part 3: User journey map co-creation

Work together to complete a full XR exercise journey map, marking key turning points, emotional changes, and innovation opportunities. This is the core output of the workshop and turns abstract user experience into a visual journey.

1. Engagement stage

The user's first experience of approaching the XR exercise system.

2. Tracking stage

Ongoing data recording and behaviour monitoring during continued use.

3. Feedback stage

How system responses and progress visualisation are presented.

Co-creation tip

Encourage participants to combine stickers, drawings, and text so the journey map is vivid and detailed. This group can choose a "journey narrator" to present the final map.

User journey mapping materials

Exercise Stage	Engagement	Tracking	Feedback
Exercise Behaviour			
Thoughts			
Touchpoints			
Emotions			
Pain Points / Challenges			
Opportunities			

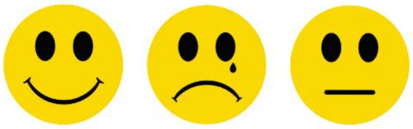

Part 4: XR co-design

Use images, videos, and other visual prompts to help participants express their ideal XR exercise experience. Participants use collage, stickers, drawings, and text to create an ideal XR scenario collage.

1. Visual prompt

Show 3–4 XR exercise images or short videos to inspire ideas.

2. Scenario selection

Participants choose preferred scenes and can combine elements from different images.

3. Open discussion

Encourage participants to share feelings and ideas about different scenes.

4. Creative collage

Use different materials to create an ideal XR exercise experience collage.

5. Presentation

Share its collage and explains the design ideas.

Five Dimensions of the Ideal XR Scenario

1. Exercise environment

The ideal exercise setting: natural scenery, familiar community spaces, a quiet room, or any other environment that feels comfortable.

2. Interactive elements

Would users like companionship? Friends, family, virtual characters, or a private space for independent exercise?

3. Type of Exercise

What kind of exercise would users like to do in the XR experience? This may include walking, tai chi, dancing, stretching, cycling, balance training, or other activities that feel suitable, enjoyable, and manageable.

4. Feedback and encouragement

What kind of feedback would users like to see? For example: “well done” prompts,

reward badges, progress icons, or voice encouragement.

5. Sensory experience

What elements could enhance immersion? For example: music, natural sounds, dynamic lighting, or even scent.

Final output

This group presents its ideal XR scenario collage, explains the design ideas and user insights, and the facilitator summarises shared needs and innovative ideas to guide future product design.

XR co-design materials

XR co-design materials included visual prompts such as photographs of different exercise environments, role stickers representing different people or companions, stickers showing different types of exercise, and examples of feedback formats, including images, charts, and interface examples. These materials were not selected based on the researchers' personal preferences. Instead, they were developed from findings generated in the earlier stages of data collection and were used to support participants in expressing their views, preferences, and ideas during the co-design process.
